# Role of digital health technologies in improving health financing and universal health coverage in Sub-Saharan Africa: a comprehensive narrative review

**DOI:** 10.3389/fdgth.2025.1391500

**Published:** 2025-05-16

**Authors:** Olutola Vivian Awosiku, Ibrahim Nafisa Gbemisola, Oluwafiponmile Thomas Oyediran, Oluwaseyi Muyiwa Egbewande, Jibril Habibah Lami, Daniel Afolabi, Melody Okereke, Fortune Effiong

**Affiliations:** ^1^ University College Hospital Ibadan, Ibadan, Nigeria; ^2^ Faculty of Clinical Sciences, University of Ilorin, Ilorin, Nigeria; ^3^ Ladoke Akintola University of Technology, Ogbomosho, Nigeria; ^4^ Faculty of Pharmaceutical Sciences, University of Ilorin, Ilorin, Nigeria; ^5^ Sub-National Support Department, Nigeria Centre for Disease Control and Prevention, Abuja, Nigeria; ^6^ Department of Pharmacognosy, Faculty of Pharmacy, University of Lagos, Yaba, Nigeria; ^7^ University of Calabar Teaching Hospital, Calabar, Nigeria

**Keywords:** UHC (universal health coverage), digital health, Africa, finacing strategy, health technologies

## Abstract

Digital technologies play a key role in developing a comprehensive and resilient healthcare delivery system in many low and middle-income countries in Sub-Saharan Africa. These technologies aim not only to address the financial accessibility gap for health needs but also to enhance innovation, partnerships, data management, and performance across healthcare stakeholders. By bridging gaps in access and reducing inequities, digital health technologies have the potential to mitigate socioeconomic disparities in healthcare delivery, particularly in resource-limited settings. This paper explores existing data on health challenges, financing, and universal health coverage in sub-Saharan Africa, along with examining digital health technologies, their adoption, and implementation. Case studies from initiatives such as M-TIBA in Kenya, JAMII in Tanzania, and L'UNION TECHNIQUE DE LA MUTUALITÉ MALIENNE in Mali are presented, along with recommendations for scale-up, policy enhancement, collaboration, support, and identification of research gaps and areas for further exploration.

## 1 Introduction

Sub-Saharan Africa (SSA) faces the dual challenge of a significant disease burden and deep-rooted socioeconomic inequalities, which digital health technologies can potentially mitigate by improving access, affordability, and equity in healthcare services. Nearly one in ten individuals experience overwhelming health costs, leading to fifteen million people falling into poverty each year due to out-of-pocket expenses (1). High-quality health services, whether for prevention or treatment, are essential for a population to attain good health and lead healthy lifestyles (2). On this basis, universal health coverage (UHC), integrated into the 2030 Agenda for Sustainable Development, is described by the World Health Organization (WHO) as ensuring that all individuals and communities can access the health services they require of satisfactory quality to be effectual, without undergoing financial distress. Addressing the urgent need for solutions is crucial, given that over a hundred million people are pushed into poverty each year due to catastrophic health expenses, especially in low- and middle-income countries (LMICs) (3). In Sub-Saharan Africa (SSA), 27 out of 48 countries experience direct out-of-pocket payments (OOPs) for healthcare services exceeding 30%.

Socioeconomic inequalities exacerbate these challenges, especially in digital health, where access to technology, literacy, and affordability play significant roles in health outcomes. Disparities in internet connectivity, mobile phone access, and digital literacy disproportionately affect rural and low-income populations, limiting the benefits of digital health interventions for those most in (4). By leveraging digital health technologies tailored to the specific needs of underserved communities, it is possible to bridge these gaps and enhance equity in healthcare access (5).

The World Health Organization (WHO) formulated a health financing framework, emphasizing integrating financing strategies into a national health policy and incorporating a service delivery plan (6). Despite ongoing international consensus regarding the importance of enhancing national health financing systems for creating sustainable and inclusive policies, health financing in low- and middle-income countries (LMICs) and individuals' access to crucial health services rely on out-of-pocket payments. These barriers to access contribute to a significant burden of preventable deaths (7). As outlined by the World Health Organization (WHO), universal health coverage (UHC) aims to transform healthcare through three key approaches (8): first, by expanding the coverage of health services; second, enhancing health service packages to address identified priorities with a focus on equity, inequalities, and ethical considerations; and last, offering affordable health insurance for everyone, thereby reducing out-of-pocket payments. This review explores how digital health technologies can enhance health financing, reduce socioeconomic inequalities, and support the achievement of Universal Health Coverage (UHC) in Sub-Saharan Africa through scalable and sustainable solutions.

Universal Health Coverage (UHC) has certain limitations and exclusions:

(i) UHC does not imply free coverage for all conceivable health interventions, irrespective of the cost. No country can sustainably offer all services free of charge. There are practical limitations to the extent of services that can be covered without jeopardizing the financial sustainability of the healthcare system. (ii) UHC is not solely focused on health financing. While financing is crucial, achieving UHC involves more than just financial considerations. It encompasses all components of the health system, including the organization and delivery of health services, ensuring accessibility, improving the quality of care, addressing social determinants of health, health technologies and communications networks, quality assurance mechanisms, governance, and legislation, etc. (iii) Beyond guaranteeing a minimal set of healthcare services, universal health care (UHC) entails gradually extending financial protection and health service coverage as additional resources become available. The objective is to gradually improve and expand health care while adjusting to the populace's changing requirements. (iv) UHC offers population-based services in addition to individual treatment programs. This includes public health campaigns, programs like fluoridating water supplies, eradicating mosquito breeding sites, and other actions to enhance population health. It strongly emphasizes providing individual and community health needs with a holistic and preventive approach to healthcare. (v) UHC encompasses much more than just health; pursuing it entails advancing equity, development goals, social inclusion, and cohesiveness (9).

## 2 Method

We conducted a narrative review of published articles on the use of digital health technologies and universal health coverage in sub-Saharan Africa with no date restrictions placed on the search. A systematic electronic search of published articles on PubMed, Scopus, Directory of Open Access Journals, Science Direct, and Google Scholar databases was conducted with the following terms: “digital health technology,” “health technology,” “universal health coverage,” “health financing,” “sub-Saharan Africa,” “Africa,” and only full papers written in the English language were reviewed for intellectual content. Papers were critically assessed, and a narrative review was conducted after data was extracted from selected papers.

## 3 Health financing in Sub-Saharan Africa

OOP payments account for more than 70% of health spending in Nigeria, well exceeding the 30% level that is advised. This rate is among the highest globally and definitely in Africa, ruining many family circles financially. After enacting the National Health Act in 2014, Nigeria made impressively but vaguely stated significant commitments to reduce OOP to guarantee citizens greater access to high-quality primary healthcare services. The National Health Act stipulates that every Nigerian is entitled to a Basic Minimum Package of Health Services (BMPHS) financed through a basic healthcare provision fund (BHCPF). This funding is to be obtained by deducting not less than 1% from the Consolidated Revenue Fund (CRF) of the Federal Government of Nigeria, as outlined in the work by (10). About half of the basic healthcare provision fund is expected to go towards paying and expanding the bare minimum package of health services, which will impact the implementation of a State Contributory Health Insurance Scheme (SCHIS), according to BHCPF expenditure strategies. As a result, the goals and objectives of the BHCPF have prompted some Nigerian states to begin drafting and implementing legislation for a state social health insurance scheme (SHIS). Over the past 20 years, the Nigerian government has experimented with several SHIS implementations. About 4% of the population was covered by the National Health Insurance Scheme (NHIS) when it was first implemented in 2000; most were federal civil service personnel (11). Nevertheless, the SHIS providers did not put much effort into making it simple for employed people in the unorganized sector to participate in and pay for health insurance. The low coverage of SHIS has been attributed to several problems, including non-acceptability and premium non-payment, particularly in the informal sector (12).

Several countries in the Sub-Saharan African (SSA) region are either in the process of or contemplating a fundamental overhaul of their healthcare financing systems to enhance health outcomes (13). The surge of enthusiasm in pursuing universal health coverage (UHC), driven by the United Nations' Sustainable Development Goal indicator 3.8, presents a distinctive chance for policymakers to leverage evidence-based academic research in crafting the most effective health systems. Numerous countries in the region have adopted or are adopting sustainable health financing strategies. These include national or social health insurance, community-based health insurance, tax-based financing, private voluntary initiatives, and micro health insurance schemes (14). However, some nations are contending with healthcare financing systems where regressive practices, like direct user fees, prevail, leading to significant out-of-pocket expenses (15). The World Health Organization regards digital technologies as a crucial facilitator for advancing the UHC agenda. It defines digital health comprehensively as “the field of knowledge and practice associated with the development and use of digital technologies to improve health.” (16).

The excitement surrounding digitization in the health sector extends to health financing, acknowledged as a pivotal aspect of health system reform for Universal Health Coverage (UHC). Mobile money services facilitating individual-level resource collection have been introduced in various countries, especially in sub-Saharan Africa (17). In addition to mobile money, Digital Health Interventions (DHIs) have been implemented, addressing the other two health financing functions, pooling and purchasing. Examples include the mobile microhealth insurance scheme, Jamii (JAMI AFRICA, 2021), and the claim management system operated by openRBF (18). Utilizing DHIs in health financing can enhance health system performance by decreasing transaction costs, facilitating strategic purchasing, and improving data-driven decision-making (18). Numerous digital Health Interventions (DHIs) lack comprehensive evaluation, and the available information is often derived from project reports rather than peer-reviewed literature, focusing predominantly on mobile money services (18). This knowledge gap raises concerns as relying on potentially flawed data from the insufficient implementation of a digital intervention might lead to unintended consequences, diverting efforts from intended health system objectives and possibly exacerbating, rather than alleviating, existing equity gaps (19).

## 4 Universal health coverage: bridging health gaps and socioeconomic inequalities in Sub-Saharan Africa

Universal Health Coverage (UHC), according to the World Health Organization (WHO), refers to the ability to ensure that all people have access to the full range of quality health services they need, when and where they need them, without experiencing financial hardship (20). Achieving UHC is one of the targets of sustainable development goals (SDGs). The progress of UHC is monitored using two indicators: coverage of essential health services (SDG 3.8.1) and catastrophic health spending (and related indicators) (SDG 3.8.2) (20). UHC embodies three main objectives: equity in access to health services, improved quality of health services, and protection against financial risks from the high cost of health services (20, 21). The goal of UHC is to guarantee that every person and community has access to high-quality healthcare without facing financial hardship. In the context of sub-Saharan Africa, where health disparities and challenges persist, the relevance of UHC cannot be overstated.

Sub-Saharan Africa (SSA) faces unique health challenges characterized by a high burden of infectious diseases such as malaria, HIV/AIDS, and tuberculosis, limited access to essential health services, and inadequate health infrastructure. UHC is relevant to sub-Saharan Africa, offering a holistic approach to address the region's health concerns. UHC can change health outcomes, promote economic growth, and enhance the general well-being of communities in the region by guaranteeing equal access to high-quality healthcare services regardless of socioeconomic background. The implementation of UHC in sub-Saharan Africa has the potential to improve health outcomes by increasing access to preventive, diagnostic, and curative services. This, in turn, can contribute to reducing the burden of infectious diseases and improving overall population health.

Achieving UHC in sub-Saharan Africa is complicated by regional variations and disparities in healthcare infrastructure, human resources, and disease burden. While several nations have achieved notable progress in broadening healthcare accessibility, others continue to face persistent challenges. The disparities can be linked to variations in the region's infrastructure for healthcare, economic development, and governance. Furthermore, many sub-Saharan health systems continue to face challenges related to high out-of-pocket healthcare costs, unequal access to care in terms of affordability, and disparities in services between socioeconomic statuses (22). These disparities are exacerbated by demographic shifts, epidemiological changes, and low GDP (23). Also, several developing countries in sub-Saharan Africa are amid multiple interrelated social, institutional, and political transitions, affecting health systems' ability to respond to UHC (21).

Implementing UHC in sub-Saharan Africa is hampered by several issues, including funding, resource development, stewardship, and service provision. Weak health systems, inadequate emergency readiness, subpar care, and insufficient medicine supply have all been identified as problems in several African nations (21). One of the main challenges in implementing UHC in sub-Saharan Africa is financing such a comprehensive healthcare system. The leading causes of financial issues are inadequate resources, ineffective money distribution, lack of health insurance coverage, fragmentation of financing structures, and inadequate funding for primary healthcare systems. According to Mills et al. (24), these financial obstacles exacerbate gaps in access to high-quality healthcare services. As a result, innovative financing mechanisms and partnerships with international organizations will be crucial in bridging the funding gap.

The development of sufficient resources, including personnel, infrastructure for healthcare, technology, and supplies, is a crucial component of UHC (25). However, there are still many obstacles to progress in sub-Saharan Africa, including a lack of technology, equipment, and materials; a shortage of financial and non-financial resources; fragmented services; unequal access to health services; a skewed distribution of available quality of care; and a lack of human resources and health workers (22). Together, these difficulties lead to unequal resource distribution, affecting the quantity and quality of services provided (25). In addition, low-quality care, increased burden of infectious and non-communicable diseases, and unregulated healthcare delivery systems make it difficult to provide services in the context of UHC. Other challenges include the rapid privatization of healthcare services, failures to expand and strengthen services, and significant healthcare inequity. These factors collectively contribute to the widening gap in healthcare access and outcomes across different regions of sub-Saharan Africa.

UHC requires robust health systems capable of delivering quality services to all. Solutions must be complemented by realistic and inventive methods considering the national, political, and economic context (25). Research indicates that to move closer to UHC, political as well as technical expertise is needed (26). Thus, to improve UHC in sub-Saharan Africa, political leaders must demonstrate a strong commitment and a clear vision to build strong institutional foundations, invest in administrative competence, and provide responsive governance (26). For UHC to be implemented successfully, the health infrastructure must be strengthened, healthcare personnel must be trained, and the medication supply chain must be improved.

To achieve UHC, UHC schemes must be carefully crafted to encompass the whole populace, focusing on the poor through customized tactics (27). To defend against financial risk, policymakers should prioritize creating and implementing national health finance systems (25). Government stewardship, stakeholder support, and equitable resource allocation must also be prioritized through suitable health funding mechanisms (25, 26). To finance the health system, adequate revenue must be generated to guarantee the effective use of money. The goal should be to decrease the percentage of the population that lacks insurance while increasing coverage for the extremely poor (26). A coherent approach that aligns all parts of health systems is imperative to effectively address core performance concerns (26). To guarantee that everyone has access to inexpensive and sustainable healthcare, an integrated dynamic UHC system that can adapt to a changing society should be adopted (27). Sub-Saharan Africa has a lot of room to grow in attaining UHC. Adopting the recommended interventions/approaches could significantly accelerate the progress toward reaching UHC in sub-Saharan Africa.

## 5 Digital health technologies: catalysts for change

Over the past three decades, diverse digital health technologies have emerged, driven by the evolution of the Internet and the World Wide Web. The journey began in the mid-1990s with the inception of informational websites, blogs, email-enabled listeners, and online discussion forums, providing a platform for laypeople to access health and medical information and share personal experiences (28).

Digital health technologies, as defined by the World Health Organization (WHO), refer to the area of expertise and application linked with advancing and utilizing digital technologies to enhance health. The scope of digital health extends beyond e-health by incorporating digital consumers and a broader array of intelligent and interconnected devices (29). It has evolved to incorporate more scientific principles and technologies, now embracing genomics, artificial intelligence, analytics, wearables, mobile applications, and telemedicine. Furthermore, these technologies are finding broader applications in medicine, extending beyond their initial roles to encompass diagnosis, treatment, clinical decision support, care management, and the delivery of healthcare services (30).

Within digital health technologies, various terms like eHealth (electronic health), telemedicine, and mHealth (mobile health) have been extensively utilized, occasionally with overlapping meanings. This highlights the need for precise scientific language that captures the subtle distinctions among these terms. In response, the term “digital health” has been coined to serve as the most comprehensive term, encompassing the application of digital technologies in the health context. While rooted in electronic health, this term extends its scope to cover related areas such as “big data” applications, genomics, and artificial intelligence.

Moreover, e-health is commonly described as utilizing information and communications technology to support health. At the same time, m-health is considered a branch of e-health focused explicitly on using wireless mobile technologies for public health. This has gained traction with the widespread adoption of smartphones and corresponding applications. On the other hand, telemedicine refers to the use of electronic communications and information technologies for the remote provision of healthcare services (31).

Leveraging digital technologies in healthcare offers numerous advantages, such as enhancing accessibility to medical services, particularly for individuals in remote locations. It also contributes to heightened safety and quality in healthcare services and products. Additionally, integrating digital tools results in enhanced knowledge and information access for healthcare professionals and communities, ultimately boosting the efficiency of the healthcare workforce (32).

Additional advantages encompass the identification and resolution of sociocultural, physical, and financial obstacles hindering fair access to healthcare and the enhancement of efficiency in the digitalization of health insurance schemes. Consequently, Digital Health (DH) promises to extend high-quality and affordable health services to the most remote areas, serving as a crucial step towards achieving Universal Health Coverage (UHC) (33). Recently, there has been a significant global push for Digital Health (DH) as a catalyst for innovation to solve these issues and accelerate the SDG and UHC goals (34). As a result, numerous DH efforts and pilot projects have been effectively carried out in numerous African nations.

The advantages mentioned, however, are only fully realized when Digital Health (DH) initiatives are harmonized with the nation's health priorities and development objectives. Optimal results are achieved when these initiatives are tailored to meet citizens' needs. Additionally, the maximum value of these benefits is unlocked when DH services are implemented on a large scale, are contextually relevant, and prove cost-effective (35).

According to De Wit et al. (36), the notable advancements in the field of ICT have made extensive implementation of Digital Health (DH) throughout Africa favorable. For example, it is estimated that around 95% of the world's population currently resides in locations well-served by mobile networks and that over seven billion mobile subscriptions—one for each person—are in use worldwide. Though less than the worldwide average, Africa's internet and mobile phone penetration rates—74% and 21.8%, respectively—remain high enough to support the expansion of distributed hosting across the continent (32).

Sub-Saharan Africa has responded well to the push to use digital health to improve service delivery. The apparent demand to develop innovative, scalable, and sustainable digital interventions to boost health systems was headed by governments, donors, corporate organizations, and academic institutions. It might be said that Sub-Saharan Africa has emerged as a leader in creating digital health initiatives to assist in providing services (37). By integrating the various parts of a working health system, digital health treatments aim to facilitate increased efficiency and transparency (38). Numerous digital interventions have been implemented in the region due to the persistent push to incorporate digital health into service delivery.

In Africa, mHealth, eLearning, telehealth, social media, and electronic health records are among the most popular digital health (DH) options (39). There are developing intergovernmental agreements between the Ministries of ICT and Health. Digital medical equipment is used in several nations to create digital patients and clinics. These clinics solve the problem of a lack of health personnel using medical equipment for remote diagnosis (40). Examples include digital ultrasonography in Tanzania, cloud-based mHealth Smart reader devices for quick diagnostic testing in Kenya, Tanzania, and Ghana, and computer-aided chest x-rays for tuberculosis diagnosis in Zambia, South Africa, and The Gambia. Furthermore, Malawi and Uganda use portable electrocardiographs that are cloud-enabled and run on smartphones (41).

The implementation of Digital Health is, however, not without challenges. African countries face challenges implementing Digital Health (DH) initiatives due to insufficient governance, institutional capacity, and funding. While many countries adopt DH strategies, slow implementation is attributed to project-based, externally funded, and unsustainable pilot projects. Unlike high-income countries with government-driven DH initiatives, African efforts often lack scalability. Uganda, for instance, imposed a moratorium on DH pilots to address this issue (42).

The discussion above has demonstrated how DH can be an instrument for bolstering health systems for universal health coverage. However, DH is not a magic bullet for achieving UHC in Africa. Additional requirements include strengthening general governance, managing health services to encourage the adoption of sustainable digital solutions, and connecting with larger government systems (32).

Universal health coverage (UHC) is described by the World Health Organisation (WHO) as “all people having access to health services they need, without encountering financial hardship, irrespective of socioeconomic status” (43).

Numerous initiatives are underway worldwide to achieve UHC. Even with a global commitment, low—and middle-income countries (LMICs), with sub-Saharan African countries contributing the largest percentage, are finding it difficult to achieve these fundamental objectives. Unaffordable healthcare delivery costs, inadequate quality, and restricted access are still significant barriers to universal access to a minimum basic package (5).

Healthcare funding in low—and middle-income countries (LMICs) is fragmented and insufficient. In sub-Saharan Africa, a small percentage of the population has health insurance (e.g., 11%–15% in Kenya), leading to high out-of-pocket expenses (36.7% of total health expenditures in 2016). In Nigeria, out-of-pocket spending reached 75.1%, causing healthcare avoidance and pushing individuals into poverty due to catastrophic expenditures (44).

To achieve efficiencies—better access, quality, and affordability—new strategies that entirely use the potential of digital and mobile technologies must be used (45). Every industry has seen a digital revolution in the past ten years; the healthcare industry is no exception. Mobile health platforms can help unify healthcare funding, reducing funding fragmentation. Funders, health managers, and legislators may find them helpful in enhancing the execution of universal health coverage initiatives in low- and middle-income nations Huisman et al. (5).

## 6 Case studies and examples

Digitization is instrumental in providing a holistic overview of healthcare system functions, improving health financing, and contributing towards universal health coverage. Digital healthcare solutions bridge access gaps, collect and pool healthcare services, and enhance overall health program efficiency. This study examines successful digital healthcare solutions in selected sub-Saharan African countries, delving into their achievements, shortcomings, insights, and lessons learned. Digital health applications in this analysis encompass various financial flows in the health system, including direct payments from consumers to health providers, health insurance premiums, demand-side incentives, conditional cash transfer (CCT), and voucher systems. These financial flows are summarized in Table 1.

**Table 1:** An overview of program initiatives and financial flows for Mali, Kenya, and Tanzania, respectively.

Country	Program	Financial flow
Mali	L’UNION TECHNIQUE DE LA MUTUALITÉ MALIENNE	Health insurance premium
Kenya	M-TIBA	Direct payments from consumers to health provider
Tanzania	JAMIE	Health insurance premium

### 6.1 Mali: L'UNION TECHNIQUE DE LA MUTUALITÉ MALIENNE

#### 6.1.1 Background

As of 2015, 4% of Mali was enrolled in Mutuelles, with a projected 40% enrollment by 2023. Mutuelles, a health premium plan, represents a key strategy for the Mali government to achieve Universal Health Coverage.

#### 6.1.2 Goals

To create a safety net for rural, low-income populations by subsidizing health insurance premiums through mobile technology.

#### 6.1.3 Stakeholders

Orange Telecommunications, The Government of Mali.

#### 6.1.4 Technology used

Orange-Cash program- The Orange-Cash program enables mobile payments for health insurance premiums, improving affordability and accessibility.

#### 6.1.5 Challenges

• Mutuelles members resisted paying “hidden” transaction fees to cover travel costs to Mutuelle facilities
• A high poverty rate hinders fee payment even with a 50% subsidy
• Lack of synergy between telecommunication service providers and UTM managers.

#### 6.1.6 Lessons learned

• Agreements with network service providers facilitate information synchronization
• Mobile money application with flexible payment options makes it easier to meet program needs
• Educating users about mobile money processes is crucial.

### 6.2 Kenya: M-TIBA

#### 6.2.1 Background

M-TIBA is a digital platform that uses mobile technology to facilitate inclusive healthcare in Kenya. The platform was launched in July 2016 and has connected thousands of patients, healthcare providers, and healthcare payers.

#### 6.2.2 Goals

To increase access to affordable quality healthcare for millions of people in Africa.

#### 6.2.3 Stakeholders

PharmAccess, Safaricom, CarePay, National Health Insurance Fund (NHIF).

#### 6.2.4 Technology used

M-TIBA platform.

#### 6.2.5 Challenges

• Limited saving funds for low-income consumers
• Intensive education is needed to enroll participants in the national health insurance
• Over-dependence on donor funding.

#### 6.2.6 Lessons learned

• Slow enrollment of new service providers requires costly training and integration processes
• Exploring integration with existing provider systems to reduce administrative burdens.

### 6.3 Tanzania: JAMII

#### 6.3.1 Background

Jamii, founded in 2016, offers small-scale health insurance services for low-income populations in the informal sector to access affordable healthcare financing schemes.

#### 6.3.2 Goals

To enable low-income populations to access affordable healthcare financing.

#### 6.3.3 Stakeholders

Jamii platform, Vodacom M-Pesa.

#### 6.3.4 Technology used

Jamii platform.

#### 6.3.5 Challenges

• Lack of financial education on micro-insurance services.

#### 6.3.6 Lessons learned

• One-on-one conversations with target customers are the way to go for grassroots activation activities and above-the-line-marketing.

## 7 Recommendations

- E-Readiness:

Digital health technology has always been with us. The emergence of the global COVID-19 pandemic has gained ground. It has come to the attention of the global world as a solution to achieving better healthcare services amid the global standstill. Africa, especially Sub-Saharan Africa, is not left out. Sub-Saharan Africa encompasses different individuals with varying belief systems, especially regarding confidential issues like their health. The openness to new methods of accessing healthcare needs to be preached from the individuals charged with delivering these various services to those to whom they will be delivered. Sensitization and awareness measures must be implemented at different levels of healthcare systems.

- Capacity building:

In light of incorporating digital health technologies into achieving universal health coverage, individuals at various delivery points must be equipped with the correct information and opportunities to properly utilize digital health technology.

- Problem centred:

Sub-Saharan African countries should look to developed countries that have succeeded in the various digital health technologies they have used. This will help reduce the research cost of making such facilities available. It also allows various research gaps to be bridged when incorporating this technology into our system. Specific solutions should be provided for specific problems for specific groups of people. It should be noted that some technologies can thrive in some countries better than others due to varying factors.

- People-centred:

Digital health technologies should not replace or erase human contact. Worthy of note is not to replace digital health technologies for human interaction. We should remember that emotional support or presence is a means of assuring patients regarding Universal Health Coverage.

- Data provision:

Sub-Saharan Africa is faced with numerous problems as regards attitudes towards healthcare. A considerable part of this is the underreporting of cases and the lack of adequate facilities. This is an area to work on to provide adequate and correct information to properly provide solutions and preventions using digital health technologies.

- Data security:

Data security is a critical challenge that must be addressed to realize the full potential of digital health technologies (46). Protecting sensitive health information is paramount to ensuring public trust and compliance with ethical and legal standards. Data breaches, cyberattacks, and inadequate infrastructure pose significant risks, especially in low-resource settings. Drawing lessons from frameworks like the Health Insurance Portability and Accountability Act (HIPAA) in the United States and the General Data Protection Regulation (GDPR) in Europe, Sub-Saharan Africa must prioritize establishing robust policies and technologies to safeguard patient data. This includes encryption, access controls, and comprehensive training for health information stakeholders. Moreover, clear legal frameworks and enforcement mechanisms must be developed to hold organizations accountable for data mishandling, thereby fostering a secure and ethical digital health ecosystem (42).

- Policies:

Developing policies to facilitate the use of digital technologies will ease the use of digital health technologies. It also provides a system for regulation.

- Incorporation into the public health system:

This involves providing an enabling environment within the healthcare system to ensure better outcomes.

- Network provision and connectivity:

Improving the network of internet service providers and providing internet connectivity even in rural areas can help ensure that every person, regardless of location, can use digital health technology.

- Alternative funding sources:

Out-of-pocket financing for healthcare services is the most widely used in many Sub-Saharan African countries. Incorporating digital health technologies into these countries can be cost-intensive, and alternative means of fund generation are needed to prevent increasing the burden of healthcare costs on citizens.

- Infrastructure & maintenance
- Monitoring & evaluation:

This is required to achieve better outcomes and help be proactive in digital health technology services, projects, and the identification of problems and the provision of solutions.

- Collaborations:

Collaboration with various corporations and countries is needed to foster digital health technology.

## 8 Conclusion

Digital health technology is an ever-evolving and continuous process that should be evidence-based and not only centered on bringing an end to a means. Universal health coverage aims to provide healthcare services that are acceptable, accessible, available, and affordable to all. In this regard, health financing is a component of achieving universal health coverage. To achieve this, it is required that all measures in providing digital health technology in Sub-Saharan Africa should avoid inequality in accessing healthcare services, as more financially advantaged individuals have better chances of affording most of these services. These services should be acquired and delivered in a manner that will reduce the cost of out-of-pocket spending on healthcare while improving health outcomes and optimizing these services.

Addressing socioeconomic inequalities is critical to realizing the full potential of digital health technologies in Sub-Saharan Africa. Disparities in digital literacy, internet connectivity, and access to mobile technologies disproportionately exclude low-income and rural populations from benefiting equally from these advancements. Incorporating tailored digital solutions that address these barriers is essential for ensuring equitable access to healthcare and reducing health disparities.

Digital health technology comes with numerous challenges, including data privacy. Adequate data protection should be ensured. Similarly, policies, frameworks, and collaborations needed to obtain this should be embraced while providing monitoring and evaluation to keep the train moving, on track, and at speed.

A Sub-Saharan Africa where digital health technology can be incorporated to achieve health financing and healthcare for all can be realized and sustained to maximum capacity.
